# Dynamic monitoring of maize grain quality based on remote sensing data

**DOI:** 10.3389/fpls.2023.1177477

**Published:** 2023-06-22

**Authors:** Weiwei Sun, Qijin He, Jiahong Liu, Xiao Xiao, Yaxin Wu, Sijia Zhou, Selimai Ma, Rongwan Wang

**Affiliations:** ^1^ College of Resources and Environmental Sciences, China Agricultural University, Beijing, China; ^2^ Collaborative Innovation Center on Forecast and Evaluation of Meteorological Disasters (CIC-FEMD), Nanjing University of Information Science and Technology, Nanjing, China

**Keywords:** maize (*Zea mays* L.), grain quality, spectral remote sensing, quality monitoring, model

## Abstract

Remote sensing data have been widely used to monitor crop development, grain yield, and quality, while precise monitoring of quality traits, especially grain starch and oil contents considering meteorological elements, still needs to be improved. In this study, the field experiment with different sowing time, i.e., 8 June, 18 June, 28 June, and 8 July, was conducted in 2018–2020. The scalable annual and inter-annual quality prediction model for summer maize in different growth periods was established using hierarchical linear modeling (HLM), which combined hyperspectral and meteorological data. Compared with the multiple linear regression (MLR) using vegetation indices (VIs), the prediction accuracy of HLM was obviously improved with the highest *R* ^2^, root mean square error (*RMSE*), and mean absolute error (*MAE*) values of 0.90, 0.10, and 0.08, respectively (grain starch content (GSC)); 0.87, 0.10, and 0.08, respectively (grain protein content (GPC)); and 0.74, 0.13, and 0.10, respectively (grain oil content (GOC)). In addition, the combination of the tasseling, grain-filling, and maturity stages further improved the predictive power for GSC (*R* ^2^ = 0.96). The combination of the grain-filling and maturity stages further improved the predictive power for GPC (*R* ^2^ = 0.90). The prediction accuracy developed in the combination of the jointing and tasseling stages for GOC (*R* ^2^ = 0.85). The results also showed that meteorological factors, especially precipitation, had a great influence on grain quality monitoring. Our study provided a new idea for crop quality monitoring by remote sensing.

## 1 Introduction

As one of the most widely grown crops in the world, maize plays an important role in the fields of food, fodder, and biofuel in a global context (Ranum et al., 2014). Approximately 72% starch, 10% protein, and 4% fat are stored in maize kernel, which provide essential nutrients and energy for people around the world (Paraginski et al., 2014). In addition, maize can be used as a raw material for food and industrial processing to produce oil, starch, sweeteners, and industrial alcohol (FAO, 2020). All the above applications pose challenges to maize quality. However, previous studies have largely focused on grain yield prediction and the response of crop growth to biotic and abiotic stresses (Vaughan et al., 2018; Pradawet et al., 2022; Shuai and Basso, 2022) studies related to maize grain quality, especially quality parameters other than protein content, have yet to be given due attention (Nuttall et al., 2017).

Accurate and timely regional crop growth monitoring and grain quality evaluation as early as possible are important directions of field management. However, the traditional way for assessing grain quality, i.e., biochemical test, though accurate, is limited by many issues, such as low efficiency, weak regional representation, and high cost. Thus, remote sensing technology and hyperspectral analysis have been widely applied to crop production estimation because of their advantages in terms of high-throughput, non-destructive, and prospective monitoring (Rodrigues et al., 2018; Xu et al., 2020; Ma et al., 2022). Crop remote sensing monitoring is realized by obtaining electromagnetic wave reflection information from the canopy through a passive sensor (Ma et al., 2022). The crop traits can be inferred by vegetation index (VI) output from remote sensing images in the visible, near-infrared, and short-wave infrared bands (Khanal et al., 2017). Moreover, the remote sensing data that indicate the growth and vitality of crop canopy can provide necessary information for estimating maize grain quality. Cho and Kang (2020) suggested that glucose produced by plant photosynthesis can be polymerized into sucrose, which is transported to the grain as the main source of starch accumulation. VIs reflecting nitrogen and chlorophyll contents of the canopy are good indicators of crop photosynthetic state (Schlemmer et al., 2013), so they can indirectly represent the level of starch accumulation. Grain oil content (GOC) can also be inferred from plant nitrogen and carbohydrate accumulation (Ghafoor et al., 2021). Grain protein formation depends on nitrogen transport and water availability, and spectral index with high sensitivity to canopy water content and nitrogen status may reflect grain protein content (GPC) effectively (Zhao et al., 2005; Ma et al., 2022). However, VIs strongly related to maize grain quality, especially grain starch content (GSC) and GOC, are not clear. In addition, with significant differences discovered across growth stages in spectral characteristics of maize (Panigrahi and Das, 2018), the determination of the optimum VIs at different stages is of importance for grain quality assessment.

The spectral information obtained by remote sensing has been used to construct the evaluation model of grain quality in recent years, mainly for GSC and GPC. In terms of the GSC prediction, Tan et al. (2011) established a direct prediction model of GSC using the structure insensitive pigment index (SIPI). The accuracy of the indirect model of GSC based on leaf nitrogen content (LNC) and SIPI was 9.7% higher than that of the direct model. Zhao et al. (2005) found that VI derived from the canopy spectral reflectance at green and red bands was significantly correlated to the final GPC. Onoyama et al. (2011) used the normalized difference vegetation index (NDVI) with partial least squares regression (PLSR) analysis to estimate GPC clearly, but they also found that prediction error increased twice or more when one prediction model used data from other years. Obviously, in the process of crop growth, VIs often cannot fully simulate the crop grain quality, and the formation of grain quality is affected by many factors (Zhang et al., 2022). Environmental factors, photosynthesis, canopy structure, and nutrient uptake directly or indirectly affect crop growth and final grain quality (Asseng and Milroy, 2006; Ning et al., 2012). Weather information, crop growth state, and their interaction should be considered in the construction of grain quality prediction models (Butts-Wilmsmeyer et al., 2019).

Crop models were considered as a way to combine the effect of different factors. For example, the leaf area index and canopy nitrogen accumulation were used to correct the DSSAT-CERES crop model, achieving high-precision prediction of GPC (Li et al., 2015). However, too many variables (meteorology, soil, management, and other input data) and complex assimilation algorithm potentially reduce predictive power and limit the large-scale application of crop models (Jin et al., 2018). Therefore, it is a new method to construct the prediction model of grain quality by synthesizing the key indices with a relatively simple algorithm. Li et al. (2020) developed an inter-annual expandable grain quality prediction model using hierarchical linear modeling (HLM), an inter-annual expandable prediction model considering the interaction between remote sensing data and meteorological data with high accuracy. HLM can incorporate hierarchical data, including nested data structures, combining multiple levels and multiple variables together (Wilson et al., 2011). In HLM, there are usually two or three levels to interpret the results of different independent variables. HLM can, therefore, not only solve regional and inter-annual crop production changes but also analyze the relationship between crop quality and remote sensing information in different regions and growth stages (Xu et al., 2020). However, the application of HLM on maize quality especially grain starch and oil prediction still needs to be improved.

Grain quality composition is the comprehensive result of various factors in crop growth stages (Dente et al., 2008; Wang et al., 2018). The factors are first reflected in the changes in growth trends with different growth periods (Li et al., 2014; Koca and Erekul, 2016) and then transformed into effects on quality. In other words, the actual growth of crops at different growth stages also contains a large amount of grain quality information (Xie et al., 2020). Therefore, based on field test data of summer maize at different sowing dates (8 June, 18 June, 28 June, and 8 July) in Gucheng Agricultural Meteorology National Observation and Research Station during 2018–2020, this study constructed monitoring models for maize grain quality (starch, protein, and oil) by HLM coupled with critical hyperspectral remote sensing data and meteorological information at different growth stages. Then, combined with different growth periods, multi-phase monitoring models were established to provide a reference for improving the monitoring capacity of grain quality.

## 2 Materials and methods

### 2.1 Field site description and experimental design

**Figure 1:**
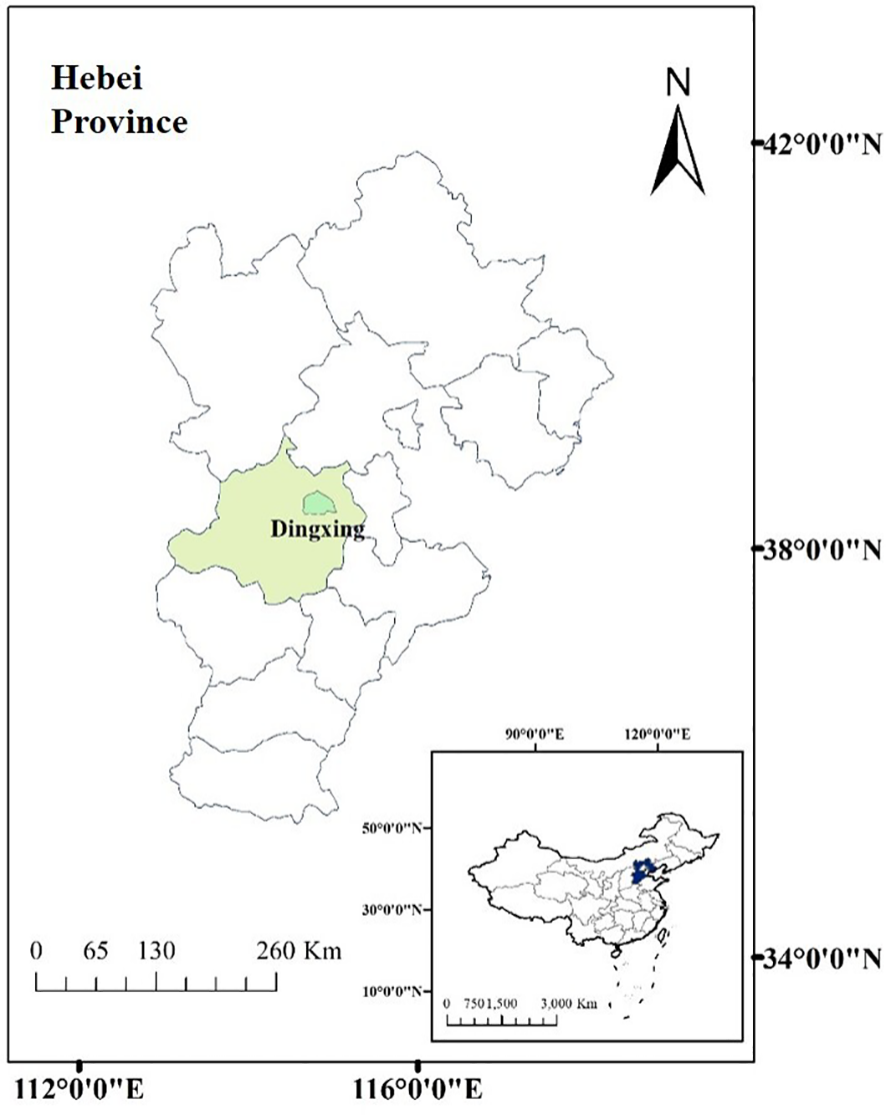
Location of study area in Dingxing County, Baoding City, Hebei Province, China.

The study was conducted at Gucheng Agricultural Meteorology National Observation and Research Station (39°08′N, 115°40′E), Dingxing County, Baoding City, Hebei Province, China (**Figure 1**). The region is located in the central part of North China, with a temperate monsoon climate. The annual mean temperature, annual mean precipitation, and annual sunshine were 12.2°C, 494 mm, and 2,403.6 h, respectively. Maize plantation is in a rotation with a previous crop of wheat in this region and surrounding area, sown in June and harvested by September–early October each year. The soil in the experimental site is characterized as sandy loam with total nitrogen at 87.00 mg kg^−1^, total phosphorus at 25.67 mg kg^−1^, total potassium at 118.55 mg kg^−1^, organic matter content at 13.67 g kg^−1^, and pH at 8.1 within a depth of 50 cm in average.

**Figure 2:**
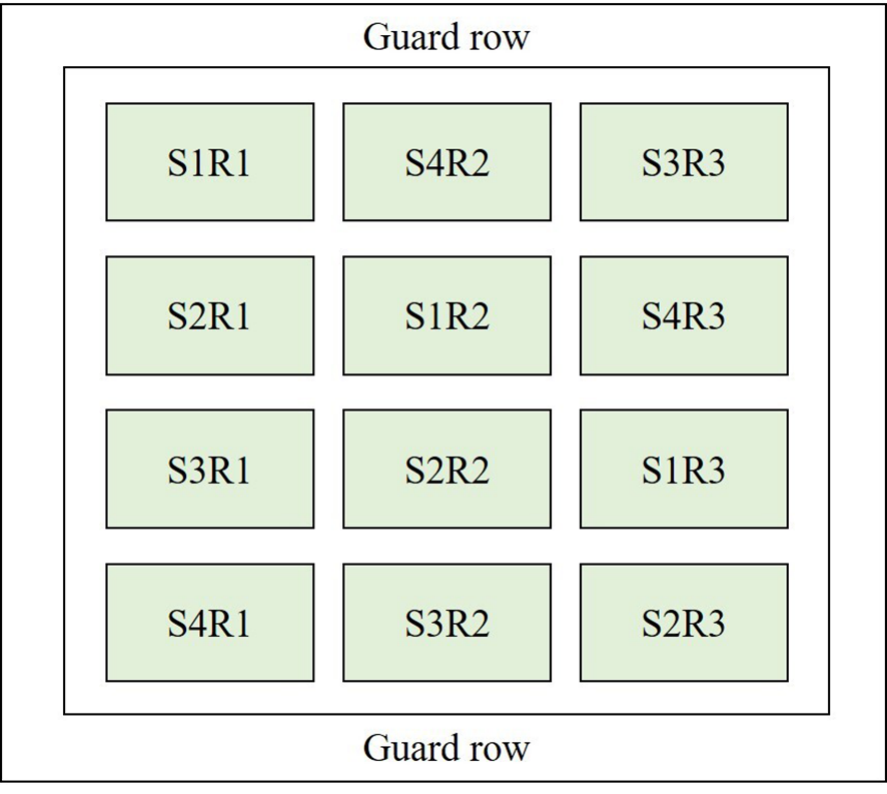
The split plot design for four sowing dates (i.e., S1, S2, S3 and S4) of maize in 2018–2020. R means repetition.

The maize hybrid Lianyu 1 was sown on 8 June (S1), 18 June (S2), 28 June (S3), and 8 July (S4) in 2018–2020. A randomized complete block design with three replications was used to arrange the treatments (**Figure 2**). The observation time of each growth stage, i.e., jointing, tasseling, grain-filling, and maturation, under different treatments, is shown in **Table 1**. Maize was planted at a density of 52 plants per plot (65,000 plants hm^−2^), with a plot dimension of 4 m × 5 m (an area of 20 m^2^). The guard row was set around the perimeter of the experimental field with a width of 2 m. Field management, including irrigation, fertilization, and pest and weed control, was carried out according to local practices.

**Table 1:** Observation time of different growth stages (i.e., jointing, tasseling, grain-filling, and maturation) under different treatments in 2018-2020.

Year	Treatment	Date				
		Sowing	Jointing	Tasseling	Grain-filling	Maturation
2018	S1	8 Jun.	3 Jul.	28 Jul.	22 Aug.	6 Sep.
	S2	18 Jun.	10 Jul.	4 Aug.	27 Aug.	12 Sep.
	S3	28 Jun.	21 Jul.	13 Aug.	5 Sep.	20 Sep.
	S4	8 Jul.	30 Jul.	22 Aug.	18 Sep.	10 Oct.
2019	S1	8 Jun.	4 Jul.	29 Jul.	21 Aug.	5 Sep.
	S2	18 Jun.	13 Jul.	5 Aug.	28 Aug.	14 Sep.
	S3	28 Jun.	20 Jul.	14 Aug.	5 Sep.	24 Sep.
	S4	8 Jul.	28 Jul.	24 Aug.	17 Sep.	10 Oct.
2020	S1	8 Jun.	4 Jul.	1 Aug.	24 Aug.	4 Sep.
	S2	18 Jun.	13 Jul.	8 Aug.	29 Aug.	14 Sep.
	S3	28 Jun.	21 Jul.	15 Aug.	4 Sep.	26 Sep.
	S4	8 Jul.	30 Jul.	26 Aug.	18 Sep.	8 Oct.

### 2.2 Canopy hyperspectral reflectance data

Canopy hyperspectral reflectance of each plot at the jointing, tasseling, grain-filling, and maturation stages of maize was collected using an ASD FieldSpec3 Spectrometer (Analytical Spectral Devices, Inc., Boulder, CO, USA). The spectral wavelength range of 350–2,500 nm was obtained by the sensor with sampling intervals of 1.38 nm for 350–1,050 nm and 2.0 nm for 1,000–2,500 nm. The spectrometer was located approximately 1.0 m above the canopy and perpendicular to the ground with a view angle of 25° so that only the plant canopy could be seen, minimizing the noise of the soil background. In addition, all observations were made under clear sky conditions between 11:30 and 14:00 for adequate light intensity. The canopy reflectance for each plot was calculated using the average of the five spectra, which was calibrated using a 0.4 m × 0.4 m BaSO_4_ calibration panel before and after measurements. The time of field canopy spectral measurements under different treatments over the years was consistent with the observation time of each growth period shown in **Table 1**.

### 2.3 Meteorological data

Meteorological data, including daily mean temperature (°C), daily precipitation (mm), and daily solar radiation (MJ m^−2^) during the summer maize growth season from 2018 to 2020, were obtained from an on-site automated weather station in Gucheng. For each phase, i.e., jointing, tasseling, grain-filling, and maturation, the effective accumulated temperature (*AT*), total precipitation (*Pre*), and total solar radiation (*Rad*) mean the sum of daily mean temperature (≥10°C), daily precipitation, and daily solar radiation from sowing to that growth period, respectively.

### 2.4 Grain quality analyses

Maize grain quality including starch, protein, and oil contents (%) was measured after harvest. GSC of dry samples was analyzed using the acid hydrolysis method in accordance with the Chinese Standard GB/T 5009.9-2016 (2016). GPC and GOC were evaluated by the macro Kjeldahl method and Soxhlet extractor method according to the National Standard of China numbered GB/T 5009.5-2016 and GB/T 5009.6-2016, respectively.

### 2.5 Selection of vegetation indices

According to the previous research, 15 VIs were considered to be potential indicators of grain quality (**Table 2**) to analyze the correlation between VIs and grain quality. Then, the optimal VIs were selected to construct the grain quality model.

**Table 2:** Selected spectral indices for predicting grain quality in this study.

Vegetation indices	Formulation	Related to	Reference
Maccioni	(R_780_ − R_710_)/(R_780_ − R_680_)	Leaf Chl	Maccioni et al., 2001
Modified simple ratio 2 (MSR2)	(R_750_/R_705_ − 1)/sqrt(R_750_/R_705 + _1)	Canopy Chl+LAI	Wu et al., 2008
Normalized difference red edge index (NDRE)	(R_790_ − R_720_)/(R_790_ + R_720_)	Canopy N	Fitzgerald et al., 2010
Normalized difference vegetation index (NDVI)	(R_800_ − R_670_)/(R_800_ + R_670_)	Canopy Chl+LAI	Marino et al., 2014
Enhanced vegetation index (EVI)	2.5 * ((R_800_ − R_670_)/(R_800_ − 6 * R_670_) − 7.5 * R_475 + _1)	Canopy Chl	Huete et al., 2002
MERIS terrestrial chlorophyll index (MTCI)	(R_750_ − R_710_)/(R_710_ − R_680_)	Canopy Chl	Dash and Curran, 2007
Photochemical reflectance index (PRI)	(R_531_ − R_570_)/(R_531_ + R_570_)	Canopy Chl	Gamon et al., 1992
Vogelmann red edge index 2 (VOG2)	(R_734_ − R_747_)/(R_715_ + R_726_)	Canopy Chl+water	Zarco-Tejada et al., 2001
Normalized difference nitrogen index (NDNI)	(log(1/R_1510_) − log(1/R_1680_))/(log(1/R_1510_) + log(1/R_1680_))	Canopy N	Serrano et al., 2002
Modified chlorophyll absorption ratio index (MCARI)	((R_750_ − R_705_) − 0.2(R_750_ − R_550_)) * (R_750_/R_705_)	Canopy Chl	Wu et al., 2008
Optimized soil-adjusted vegetation index (OSAVI)	1.16(R_800_ − R_670_)/(R_800_ + R_670 + _0.16)	Canopy Chl+LAI	Rondeaux et al., 1996
Normalized pigment chlorophyll index (NPCI)	(R_680_ − R_430_)/(R_680_ + R_430_)	Canopy Chl+N	Peñuelas et al., 1994
Normalized difference water index (NDWI)	(R_860_ − R_1240_)/(R_860_ + R_1240_)	Canopy Water	Gao, 1996
Structure insensitive pigment index (SIPI)	(R_800_ − R_445_)/(R_800_ − R_680_)	Canopy Chl+Car	Peñuelas et al., 1995
Ratio vegetation index (RVI)	R_870_/R_660_	Canopy Chl+LAI	Zhu et al., 2008

### 2.6 Grain quality prediction model

#### 2.6.1 Missing data imputation

The k-nearest neighbor (kNN) method was applied to impute the missing value for the sample at the tasseling stage under S2 treatment in 2018. To impute a missing value for a target sample, find the k most similar samples according to the defined distance measure (calculated using VI values that exist in the target sample and candidate neighbor sample) (Kim et al., 2005).

#### 2.6.2 Multiple linear regression

First, a multiple linear regression equation (Eq. 1) was used to establish a grain quality prediction model based on the vegetation index:


(1) $$G=k+k_{1}\cdot VI_{1}+k_{2}\cdot VI_{2}+\epsilon$$

where *G* means measured grain quality, and *VI*_1_ and *VI*_2_ mean the two vegetation indices with the strongest correlation with grain quality under different phases. In addition, *k*, *k*_1_, and *k*_2_ represent the constant terms and the parameter terms for the corresponding variable of the linear model, and *ϵ* is the error term.

#### 2.6.3 Gray relation analysis

Gray relation analysis (GRA) was used for indefinite relation between characteristic variables (i.e., GSC, GPC, and GOC) and independent variables (i.e., VIs, *AT*, *Pre*, and *Rad*). The main procedure of GRA is first to normalize the values of all variables to produce a comparable sequence, which is called gray relation generation. From these sequences, the reference sequence (characteristic variable) is defined. Then, the gray relation coefficients between all comparable sequences and reference sequences are calculated. Finally, based on these gray relation coefficients, the gray relation level represented by the relevant degree (RD) between the reference sequence and each comparable sequence is calculated.

#### 2.6.4 Hierarchical linear modeling

Furthermore, in order to consider the influence of inter-annual environmental factors on grain quality, HLM is a least squares regression analysis method that considers the nested structure of data, which has begun to be applied to yield and the GPC prediction in recent years (Li et al., 2020; Xu et al., 2020). The GSC and GOC are also affected by nitrogen and carbon metabolism, which can be reflected by the maize canopy spectrum (Yue et al., 2022; Li et al., 2023). Thus, HLM was applied to estimate the starch and oil contents in this study. HLM can stratify the dataset to comprehensively analyze the relationship between the data within the layer (the first-layer model) and the data outside the layer (the second-layer model) in view of the independence between the data. In this study, the first-layer model (*L*1) was a quality prediction model from the canopy spectra:


(2) $$L1:\;G=\beta_{0j}+\beta_{1j}\cdot VI_{1}+\beta_{2j}\cdot VI_{2}+r_{ij}$$

where *β*_0j_, *β*_1j_, and *β*_2j_ represent the intercept and coefficients of *VI*_1_ and *VI*_2_ under different phases in *L*1, respectively, and *r*_ij_ represents the random error.

The second layer model (*L*2) is based on the normalized value of the model coefficient in the *L*1 and the external meteorological data (*AT*, *Pre*, and *Rad*), as follows:


(3) $$L2:\;\beta_{nj}=\gamma_{n0}+\gamma_{n1}\cdot AT+\gamma_{n2}\cdot Rad+\gamma_{n3}\cdot Pre+\mu_{nj}$$

where *β*_nj_ means the intercept and slope of *L*1; *γ*_n0_ is the intercept of *L*2; and *γ*_n1_, *γ*_n2_, and *γ*_n3_ are the model coefficients of *AT*, *Rad*, and *Pre* in *L*2, respectively; *μ*_nj_ is the random error.

#### 2.6.5 Combination of prediction model under different phases

In this research, the stepwise multiple regression method was used for the combination of prediction models under different phases to improve prediction power. In order to avoid the possibility of multicollinearity among independent variables, we first tested the variance inflation factor (VIF) of each variable in the equation and then obtained the optimal regression equation by stepwise multiple regression analysis. Bidirectional elimination is applied to test the variables to be included or excluded at each step. From all the independent variables available for selection, the variables that have a significant impact on the dependent variable are selected to establish the regression equation, while variables with no significant effect on the dependent variable were not added to the equation. The equation was expressed as follows:


(4) $$G=\beta_{0}+\beta_{1}G_{1}+\beta_{2}G_{2}+\cdot \cdot \cdot +\beta_{n}G_{n}+\epsilon$$

where *β*_0_ is a constant; *G*_1_, *G*_2_, …, *G*_n_ mean the optimal independent variables (predicted value under different phases) for the model; *β*_1_, *β*_2_, …, *β*_n_ are the regression coefficients corresponding to the predicted value in different phases; *ϵ* is the error term.

### 2.7 Statistical analysis

Pearson’s correlation coefficient (*r*) between vegetation indices and grain quality (GSC, GPC, and GOC) were analyzed using IBM SPSS Statistics 24.0 (IBM Corp., Armonk, NY, USA). In all prediction models, 80% of the dataset was the modeling set, 20% was the validation set, and the random state was set to 42 to ensure the consistency of dataset partitioning at different phases. To test the performance of different prediction models, the coefficient of determination (*R* ^2^) (Eq. 5), root mean square error (*RMSE*) (Eq. 6), and mean absolute error (*MAE*) (Eq. 7) were used as measures of accuracy.


(5) $$R^{2}=\frac{\sum_{i=1}^{n}\left(Y_{i}-Y_{i}^{'}\right)}{\sum_{i=1}^{n}\left({\hat{Y}}_{i}-Y_{i}\right)}$$

(6) $$RMSE=\sqrt{\frac{1}{n}\sum_{i=1}^{n}{\left(Y_{i}-Y_{i}^{'}\right)}^{2}}$$

(7) $$MAE=\frac{\sum_{i=1}^{n}\left|Y_{i}-Y_{i}^{\;\prime }\right|}{n}$$

where *n* is the number of observations; *Y_i_* and *Y_i_’* are the *i*th measured and simulated data, respectively; *Ŷ* is the mean value of measured data.

All statistical indicators were calculated using python 3.9 (Python Software Foundation, Portland, OR, USA), and all figures were drawn by Microsoft Office Excel (Microsoft Corporation, USA) and the ggplot package of R language (RStudio Inc., Boston, MA, USA).

## 3 Results

### 3.1 Correlation between VIs and grain quality

**Figure 3:**
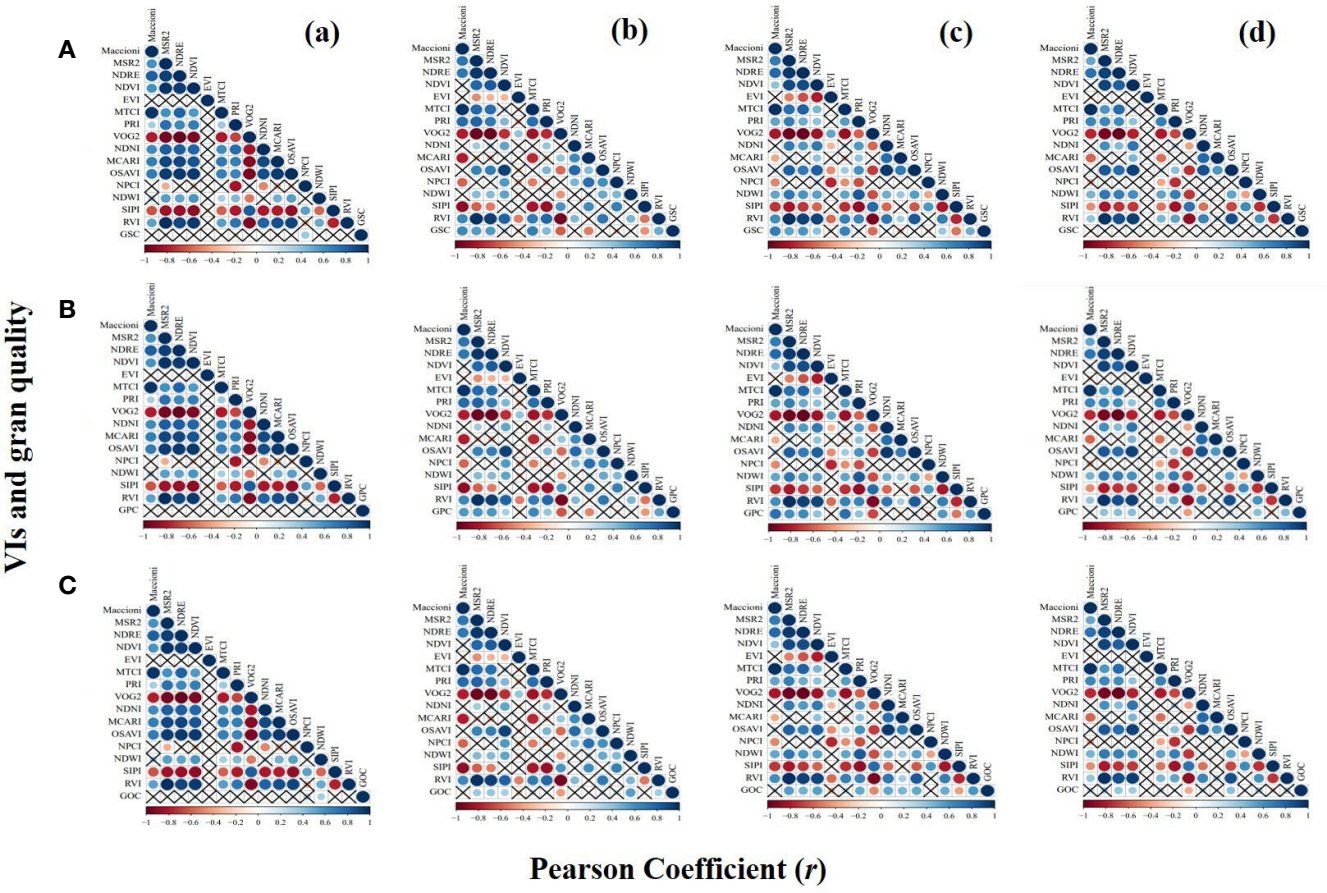
Correlations between VIs and grain quality, i.e., **(A)** GSC, **(B)** GPC, and **(C)** GOC under different phases, i.e., **(a)** jointing stage, **(b)** tasseling stage, **(c)** grain-filling stage, and **(d)** maturation stage. × indicates no significant correlation. The larger the circle, the stronger the correlation. VIs, vegetation indices; GSC, grain starch content; GPC, grain protein content; GOC, grain oil content.

The correlation between VIs calculated by the calibration dataset and grain quality under different phases is depicted in **Figure 3**. For GSC, only normalized pigment chlorophyll index (NPCI) was significantly correlated with GSC at the jointing stage. However, 60% VIs were significantly correlated with GSC at the tasseling and grain-filling stages. No significant correlation was found between VIs and GSC at the maturation stage. For GPC, VIs had no significant correlation with GPC at the jointing stage. Approximately 67% VIs were significantly correlated with GPC at the tasseling and grain-filling stages. In addition, 40% VIs showed a significant correlation with GPC at the maturation stage. In particular, VIs that showed a significant correlation with GSC and GPC at the tasseling and grain-filling stages had an extreme consistency. For GOC, VIs at the jointing stage had no significant correlation with GOC except normalized difference water index (NDWI). At the tasseling stage, 40% VIs were significantly correlated with GOC. At the grain-filling stage, 73% VIs were significantly correlated with GOC, while only 27% VIs were significantly correlated with GOC at the maturation stage.

The relationship between grain quality and the two VIs with the highest correlation at different growth stages can be seen in **Table 3**. VIs had no significant correlation with GSC, GPC, and GOC at the jointing stage (*p*< 0.01), except for NPCI with GSC (*r* = 0.37) and NDWI with GOC (*r* = 0.33). All selected best two VIs showed a highly significant correlation with GSC, GPC, and GOC at the tasseling and grain-filling stages (*p*< 0.01), with MERIS terrestrial chlorophyll index (MTCI) (*r* = 0.69), MTCI (*r* = 0.64), and NDWI (*r* = 0.55) showing the best correlation with GSC, GPC, and GOC, respectively, at the tasseling stage. However, Maccioni (*r* = 0.69), MTCI (*r* = 0.74), and ratio vegetation index (RVI) (*r* = 0.58) had a higher significant correlation (*p*< 0.01) with GSC, GPC, and GOC at the grain-filling stage. However, NDWI and NDVI had no significant correlation with GSC at the maturation stage, while RVI and Vogelmann red edge index 2 (VOG2) (*r* = 0.44 and −0.43, respectively) and optimized soil-adjusted vegetation index (OSAVI) and normalized difference red edge index (NDRE) (*r* = 0.41 and 0.39, respectively) showed a significant correlation with GPC and GOC, respectively.

**Table 3:** Correlation coefficients between the two best VIs and grain quality (i.e., GSC, GPC, and GOC) under different phases.

Phases	VI	GSC	VI	GPC	VI	GOC
Jointing	NPCI	0.37**	NPCI	0.29	NDWI	0.33**
	PRI	−0.21	NDWI	0.18	NPCI	0.30
Tasseling	MTCI	0.69**	MTCI	0.64**	NDWI	0.55**
	Maccioni	0.63**	VOG2	−0.63**	VOG2	−0.43**
Grain-filling	Maccioni	0.69**	MTCI	0.74**	RVI	0.58**
	MTCI	0.67**	Maccioni	0.74**	MSR2	0.55**
Maturation	NDWI	0.25	RVI	0.44**	OSAVI	0.41**
	NDVI	0.21	VOG2	−0.43**	NDRE	0.39**

VIs, vegetation indices; GSC, grain starch content; GPC, grain protein content; GOC, grain oil content; NPCI, normalized pigment chlorophyll index; NDWI, normalized difference water index; PRI, photochemical reflectance index; MTCI, MERIS terrestrial chlorophyll index; VOG2, Vogelmann red edge index 2; RVI, ratio vegetation index; MSR2, modified simple ratio 2; NDVI, normalized difference vegetation index; OSAVI, optimized soil-adjusted vegetation index; NDRE, normalized difference red edge index.

**Indicates extremely significant correlation (p< 0.01).

### 3.2 Grain quality prediction model using MLR

**Figure 4:**
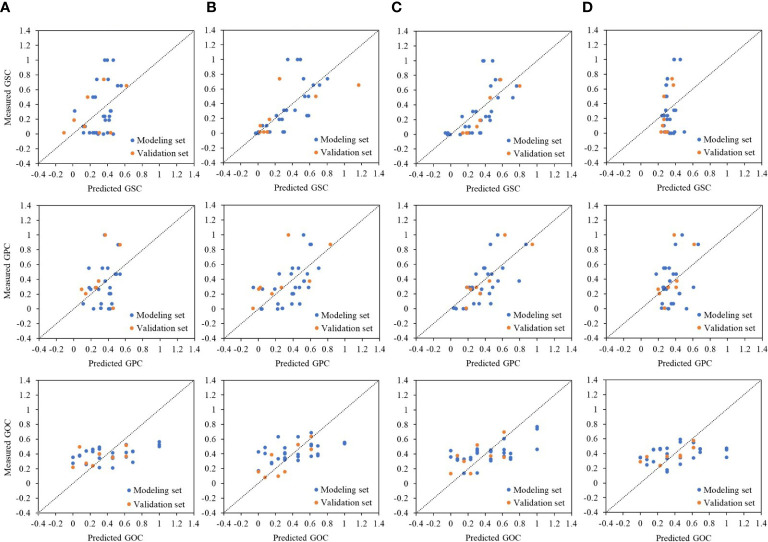
Relationships between measured and predicted grain quality, i.e., GSC, GPC, and GOC under different phases, i.e., **(A)** jointing stage, **(B)** tasseling stage, **(C)** grain-filling stage, and **(D)** maturation stage, by MLR method. The dashed line represents the 1:1 line. GSC, grain starch content; GPC, grain protein content; GOC, grain oil content; MLR, multiple linear regression.

The VIs shown in **Table 3** were used to construct the prediction model under different phases. For the GSC, GPC, and GOC prediction, the bad *R* ^2^ was found in the jointing and maturation stages. The multiple linear regression (MLR) model for the GSC prediction had *R* ^2^, *RMSE*, and *MAE* values of 0.49, 1.28%, and 0.84%, respectively, in the tasseling stage, and 0.53, 1.23%, and 0.91%, respectively, in the grain-filling stage (both using Maccioni and MTCI); the best prediction occurred in the tasseling stage because of the overfitting based on the validation set in the grain-filling stage. For the GPC prediction, the best accuracy was found in the grain-filling stage with *R* ^2^, *RMSE*, and *MAE* values of 0.49, 0.56%, and 0.42%, respectively. For the GOC prediction, the accuracy of the MLR decreased further with an *R* ^2^ of 0.36 in the tasseling stage and 0.30 in the grain-filling stage (**Table 4**). For GSC and GPC, low prediction power was associated with a smaller fluctuation range of predicted value than the measured value, while it reversed for the GOC prediction (**Figure 4**). MLR model constructed by VIs cannot simulate the variability of factors other than crop canopy traits, leading to poor prediction accuracy.

**Table 4:** Formula and statistical data for predicting grain quality (i.e., GSC, GPC, and GOC) under different phases by MLR model.

Grain quality	Phases	Formula	Modeling set			Validation set		
			*R* ^2^	*RMSE*%	*MAE*%	*R* ^2^	*RMSE*%	*MAE*%
GSC	Jointing	64.38 + 32.16 * PRI + 11.73 * NPCI	0.11	1.70	1.55	0.27	1.75	1.55
	Tasseling	102.10 − 56.76 * Maccioni + 2.06 * MTCI	0.49	1.28	0.84	0.38	1.60	1.09
	Grain-filling	50.70 + 10.97 * Maccioni + 1.33 * MTCI	0.53	1.23	0.91	0.11	1.92	1.61
	Maturation	65.80 − 0.55 * NDVI + 9.54 * NDWI	0.06	1.74	1.41	0.06	1.97	1.53
GPC	Jointing	7.16 + 3.05 * NPCI + 7.47 * NDWI	0.14	0.72	0.57	0.06	0.81	0.60
	Tasseling	5.71 + 0.19 * MTCI − 2.98 * VOG2	0.47	0.57	0.47	0.24	0.73	0.52
	Grain-filling	−0.99 + 8.68 * Maccioni + 0.38 * MTCI	0.49	0.56	0.42	0.75	0.42	0.32
	Maturation	7.21 − 0.88 * VOG2 + 0.11 * RVI	0.14	0.72	0.60	0.34	0.68	0.46
GOC	Jointing	3.17 + NPCI + 3.40 * NDWI	0.15	0.28	0.24	0.24	0.42	0.38
	Tasseling	2.63 − 0.16 * VOG2 + 5.75 * NDWI	0.36	0.24	0.20	0.20	0.44	0.39
	Grain-filling	3.06 − 0.20 * MSR2 + 0.14 * RVI	0.30	0.25	0.22	0.38	0.38	0.32
	Maturation	2.49 + 0.06 * NDRE + 1.85 * OSAVI	0.21	0.27	0.23	0.04	0.48	0.41

GSC, grain starch content; GPC, grain protein content; GOC, grain oil content; MLR, multiple linear regression; NPCI, normalized pigment chlorophyll index; NDWI, normalized difference water index; PRI, photochemical reflectance index; MTCI, MERIS terrestrial chlorophyll index; VOG2, Vogelmann red edge index 2; RVI, ratio vegetation index; MSR2, modified simple ratio 2; NDVI, normalized difference vegetation index; OSAVI, optimized soil-adjusted vegetation index; NDRE, normalized difference red edge index; RMSE, root mean square error; MAE, mean absolute error.

### 3.3 Relevant degree between VIs, meteorological factors, and grain quality using GRA

Different from correlation analysis, which was used to determine the strength of linear relationships between factors, GRA can be used to comprehensively describe the importance of different factors to the target value. The results showed that meteorological factors, i.e., *AT*, *Rad*, and *Pre*, had a strong relation to GSC, especially the RD between accumulated precipitation, and GSC ranked first in all stages (RD = 0.71–0.77). The most noteworthy was the total precipitation during the maize growth period (sowing–maturation), giving the strongest relevant degree with GSC (RD = 0.75), followed by total accumulated temperature (RD = 0.61) and total radiation (RD = 0.60). Meteorological factors showed weak RD with GPC in all stages except for total precipitation from sowing to maturation (RD = 0.72, rank second). Compared to meteorological factors, VIs had a stronger relevant degree with GOC in all stages except the early growth stage (sowing–jointing) (**Table 5**).

**Table 5:** Relevant degree between VIs, meteorological factors (i.e., *AT*, *Rad*, and *Pre*), and grain quality (i.e., GSC, GPC, and GOC) using GRA.

Grain quality	Jointing			Tasseling			Grain-filling			Maturation		
	Object	RD	Rank	Object	RD	Rank	Object	RD	Rank	Object	RD	Rank
GSC	PRI	0.59	5	Maccioni	0.62	4	Maccioni	0.72	3	NDVI	0.56	5
	NPCI	0.64	4	MTCI	0.71	2	MTCI	0.77	2	NDWI	0.59	4
	AT	0.68	2	AT	0.65	3	AT	0.66	4	AT	0.61	2
	Rad	0.65	3	Rad	0.59	5	Rad	0.62	5	Rad	0.60	3
	Pre	0.71	1	Pre	0.77	1	Pre	0.77	1	Pre	0.75	1
GPC	NPCI	0.67	1	MTCI	0.73	1	Maccioni	0.72	2	VOG2	0.55	5
	NDWI	0.59	5	VOG2	0.60	4	MTCI	0.74	1	RVI	0.72	1
	AT	0.66	2	AT	0.66	2	AT	0.69	3	AT	0.68	3
	Rad	0.66	3	Rad	0.59	5	Rad	0.59	5	Rad	0.63	4
	Pre	0.65	4	Pre	0.64	3	Pre	0.67	4	Pre	0.72	2
GOC	NPCI	0.62	5	VOG2	0.66	4	MSR2	0.70	2	NDRE	0.66	1
	NDWI	0.66	3	NDWI	0.72	1	RVI	0.71	1	OSAVI	0.65	2
	AT	0.75	1	AT	0.69	2	AT	0.68	3	AT	0.59	5
	Rad	0.66	2	Rad	0.65	5	Rad	0.62	4	Rad	0.62	3
	Pre	0.63	4	Pre	0.66	3	Pre	0.61	5	Pre	0.61	4

VIs, vegetation indices; GSC, grain starch content; GPC, grain protein content; GOC, grain oil content; GRA, Gray relation analysis; RD, relevant degree; NPCI, normalized pigment chlorophyll index; NDWI, normalized difference water index; PRI, photochemical reflectance index; MTCI, MERIS terrestrial chlorophyll index; VOG2, Vogelmann red edge index 2; RVI, ratio vegetation index; MSR2, modified simple ratio 2; NDVI, normalized difference vegetation index; OSAVI, optimized soil-adjusted vegetation index; NDRE, normalized difference red edge index.

### 3.4 Grain quality prediction model using HLM

**Figure 5:**
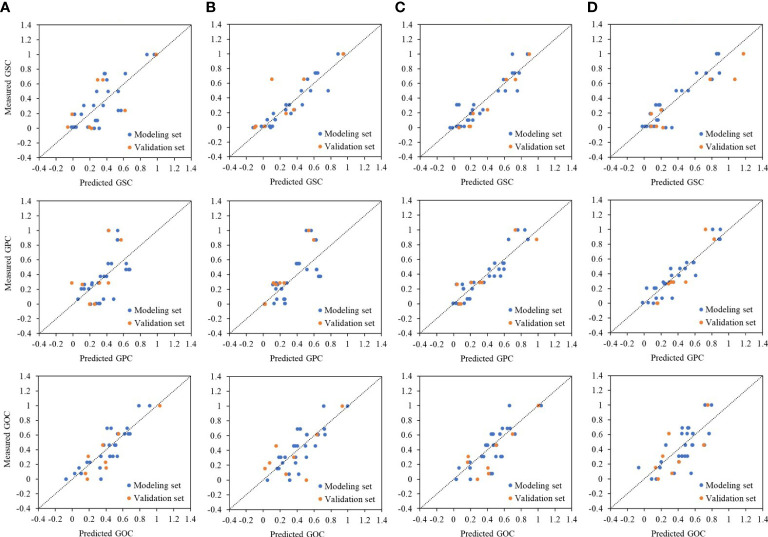
Relationships between measured and predicted grain quality, i.e., GSC, GPC, and GOC, under different phases, i.e., **(A)** jointing stage, **(B)** tasseling stage, **(C)** grain-filling stage, and **(D)** maturation stage by HLM method. The dashed line represents the 1:1 line. GSC, grain starch content; GPC, grain protein content; GOC, grain oil content; HLM, hierarchical linear modeling.

**Figure 6:**
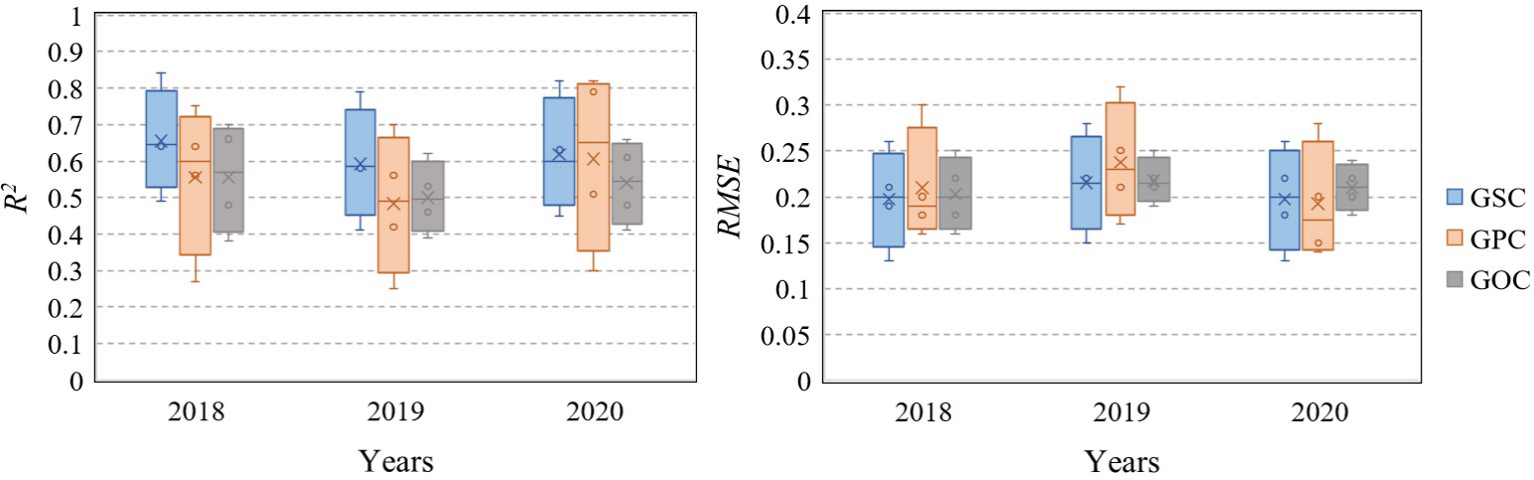
Boxplots comparing prediction performance of HLM under different years, i.e., 2018, 2019, and 2020, in different phases, i.e., jointing, tasseling, grain-filling, and maturation, by using *R* ^2^ and *RMSE*. HLM, hierarchical linear modeling; RMSE, root mean square error.

As shown in **Table 5**, meteorological factors had a correlation with grain quality, especially GSC. Therefore, meteorological factors should be taken into account in the grain quality prediction model using HLM. The two VIs that had the highest correlation with quality were also applied in the construction of the quality prediction model for comparison with MLR. The parameters in **Table 6**, i.e., *γ_n_*_1_, *γ_n_*_2_, and *γ_n_*_3_, explain the contribution of *AT*, *Rad*, and *Pre*, respectively. For the GSC prediction, the high accuracy of the model was realized in the middle and late growth stages with *R* ^2^, *RMSE*, and *MAE* values of 0.90, 0.10, and 0.08, respectively (from sowing to tasseling); 0.85, 0.12, and 0.09, respectively (from sowing to grain-filling); and 0.85, 0.12, and 0.10, respectively (from sowing to maturation). For the GPC prediction, the model in the later growth stage performed better, with *R* ^2^, *RMSE*, and *MAE* values of 0.84, 0.11, and 0.10, respectively (from sowing to grain-filling), and 0.87, 0.10, and 0.08, respectively (from sowing to maturation). The GSC and GPC models had certain similar rules, but the GOC model was completely different with the best *R* ^2^, *RMSE*, and *MAE* values of 0.74, 0.13, and 0.10, respectively, at the early growth stage from the sowing to the jointing stage. The GOC model in the middle and late growth stages showed poor predictive ability (**Table 7**). Compared with the MLR method, the HLM method showed significant improvement in the prediction of GSC, GPC, and even GOC under different phases (**Figure 5**). In addition, the HLM model generally performed well over the respective years (2018–2020), with *R* ^2^ varying from 0.79 to 0.84 and *RMSE* from 0.13 to 0.15 at the grain-filling stage for GSC estimation, and *R* ^2^ varying from 0.70 to 0.82 and *RMSE* from 0.14 to 0.17 at the grain-filling and maturation stages for GPC estimation. For the evaluation of GOC, the highest *R* ^2^ varied from 0.62 to 0.70 and the lowest *RMSE* from 0.16 to 0.19 at the jointing stage. In general, the prediction effect of HLM in 2018 and 2020 was slightly better than in 2019, with *R* ^2^ improved by 0.03 and 0.05 (GSC), 0.05 and 0.09 (GPC), and 0.08 and 0.04 (GOC) (**Figure 6**).

**Table 6:** Coefficient value of each variable in grain quality (i.e., GSC, GPC and GOC) prediction model by HLM method under different phases.

Phases	Fixed effect	GSC				GPC				GOC			
		*γ_n_*_0_	*γ_n_*_1_	*γ_n_*_2_	*γ_n_*_3_	*γ_n_*_0_	*γ_n_*_1_	*γ_n_*_2_	*γ_n_*_3_	*γ_n_*_0_	*γ_n_*_1_	*γ_n_*_2_	*γ_n_*_3_
Jointing	For intercept, *β*_0_	−0.39	−3.82	4.05	1.60	0.32	0.71	−1.63	−0.15	−0.88	−4.12	5.41	2.97
	For *VI*_1_ slope, *β*_1_	0.78	4.35	−4.86	−2.61	−0.43	0.69	0.62	0.44	2.35	2.41	−5.19	−3.43
	For *VI*_2_ slope, *β*_2_	0.41	4.97	−5.20	−0.65	0.46	−0.09	0.23	−0.81	−0.28	5.39	−4.67	−1.52
Tasseling	For intercept, *β*_0_	−2.79	−9.67	15.76	−1.90	−0.57	−0.10	1.04	1.73	−1.92	2.52	−0.08	1.81
	For *VI*_1_ slope, *β*_1_	3.73	41.19	−55.65	11.19	1.07	2.11	−3.07	−1.90	1.23	−5.51	2.84	0.79
	For *VI*_2_ slope, *β*_2_	0.86	−37.97	46.11	−10.96	0.15	0.70	−0.86	−0.83	2.55	0.64	−2.53	−2.80
Grain-filling	For intercept, *β*_0_	−0.32	−0.07	0.14	2.46	−0.05	1.15	−1.14	1.04	−0.21	−0.78	1.22	1.20
	For *VI*_1_ slope, *β*_1_	−0.12	−9.98	9.65	−2.19	−1.00	−22.64	25.76	3.50	−9.36	−10.09	22.15	12.26
	For *VI*_2_ slope, *β*_2_	1.87	13.41	−13.77	−1.78	2.03	30.16	−34.49	−6.84	10.36	13.34	−25.94	−14.76
Maturation	For intercept, *β*_0_	−0.19	−0.38	0.61	2.14	−0.14	1.37	−1.78	2.24	0.56	1.17	−1.83	−0.28
	For *VI*_1_ slope, *β*_1_	−0.46	0.88	0.09	−0.82	−0.52	−2.36	3.93	−0.50	0.02	5.13	−3.74	−2.44
	For *VI*_2_ slope, *β*_2_	0.63	−0.13	−0.79	−1.18	0.46	3.00	−2.28	−3.53	0.39	−3.47	2.55	1.32

VI_1_ and VI_2_ are the VIs corresponding to each phase in **Table 3**; the absolute value of the correlation coefficient of VI_1_ is larger than that of VI_2_.

GSC, grain starch content; GPC, grain protein content; GOC, grain oil content; HLM, hierarchical linear modeling; VIs, vegetation indices.

**Table 7:** Statistical data for predicting grain quality (i.e., GSC, GPC, and GOC) under different phases using HLM.

Grain quality	Phases	Modeling set			Validation set		
		*R* ^2^	*RMSE*	*MAE*	*R* ^2^	*RMSE*	*MAE*
GSC	Jointing	0.63	0.19	0.15	0.50	0.25	0.22
	Tasseling	0.90	0.10	0.08	0.61	0.22	0.15
	Grain-filling	0.85	0.12	0.09	0.90	0.11	0.10
	Maturation	0.85	0.12	0.10	0.69	0.19	0.16
GPC	Jointing	0.35	0.23	0.18	0.30	0.29	0.24
	Tasseling	0.49	0.21	0.17	0.64	0.21	0.15
	Grain-filling	0.84	0.11	0.10	0.84	0.14	0.11
	Maturation	0.87	0.10	0.08	0.82	0.15	0.12
GOC	Jointing	0.74	0.13	0.10	0.79	0.14	0.12
	Tasseling	0.65	0.16	0.12	0.42	0.23	0.18
	Grain-filling	0.62	0.16	0.12	0.62	0.19	0.15
	Maturation	0.50	0.19	0.16	0.53	0.21	0.19

GSC, grain starch content; GPC, grain protein content; GOC, grain oil content; HLM, hierarchical linear modeling; RMSE, root mean square error; MAE, mean absolute error.

### 3.5 Combination of grain quality prediction model under different growth stages

To further improve the prediction accuracy of grain quality, we combined the predicted values using the HLM method under different growth stages through multiple stepwise regression equations to obtain the best models (**Table 8**). For the GSC prediction, the best model was produced by the combination of the predicted value under three stages, i.e., tasseling, grain-filling, and maturation stages, with *R* ^2^, *RMSE*, and *MAE* values of 0.96, 0.06, and 0.05, respectively. For the GPC and GOC prediction, the best model was the combination of two growth stages. The difference is that the best GPC prediction model was the combination of the grain-filling and maturation stages with *R* ^2^, *RMSE*, and *MAE* values of 0.90, 0.09, and 0.07, respectively, while the best GOC prediction model was the combination of the jointing and tasseling stages with *R* ^2^, *RMSE*, and *MAE* values of 0.85, 0.10, and 0.08, respectively.

**Table 8:** Best prediction models based on different multiple growth period combinations with model evaluation and verification.

Model type	Formula	Modeling set			Validation set		
		*R* ^2^	*RMSE*	*MAE*	*R* ^2^	*RMSE*	*MAE*
GSC	−0.021 + 0.42 * *G*_2 + _0.17 * *G*_3 + _0.48 * *G*_4_	0.96	0.06	0.05	0.94	0.09	0.06
GPC	−0.02 + 0.45 * *G*_3 + _0.61 * *G*_4_	0.90	0.09	0.07	0.87	0.12	0.10
GOC	−0.10 + 0.68 * *G*_1 + _0.55 * *G*_2_	0.85	0.10	0.08	0.77	0.15	0.11

G_1_, G_2_, G_3_, and G_4_ mean predicted values under different phases, i.e., jointing, tasseling, grain-filling, and maturation, respectively.

RMSE, root mean square error; MAE, mean absolute error; GSC, grain starch content; GPC, grain protein content; GOC, grain oil content.

## 4 Discussion

### 4.1 Prediction model of GSC, GPC, and GOC

In this study, the VIs that significantly correlated with GSC and GPC were basically the same at the tasseling and grain-filling stages. Maccioni and MTCI showed the strongest correlation with GPC and GOC in all phases (**Table 3**). These results indicated that the spectral bands monitoring GSC and GPC were similar, and in addition, chlorophyll level had a great influence on GSC and GPC. With the growth process, the correlation between VIs and grain quality started to increase in the jointing stage, reached the plateau in the tasseling and grain-filling stages, and declined in the maturation stage. The high correlation between VIs and grain quality might be related to the accurate vegetation index caused by the fully extended leaves, high coverage, and mature canopy at the tasseling and grain-filling stages. However, a weak correlation was indicated at the jointing and maturation stages with low vegetation coverage and chlorophyll content (Xie et al., 2020). In addition, the correlation between VIs and GSC or GPC was significantly stronger than that between VIs and GOC, which was related to the fact that the VIs mainly reflected the information of canopy chlorophyll and nitrogen contents and could be used to predict GSC and GPC directly.

However, poor prediction accuracy is shown in the MLR model with only VIs (**Table 4**), which is also stated by Xue et al. (2007) and Chen (2020). VI, which represented the canopy state, is a key and direct indicator for grain yield prediction (Kuri et al., 2014), while an indirect relationship is known between VIs and grain quality. Grain quality is affected by many factors, such as hybrid characteristics, agronomic practices, and weather information (Gooding, 2017). Although the genotype has a great influence on the final grain quality composition, the temperature, water, and other conditions during the whole crop growth stage, especially the meteorological factors during the critical growth period, also play a certain role in the formation of grain quality (Butts-Wilmsmeyer et al., 2019). Therefore, the environmental conditions at different growth stages were fully considered in this study, and remote sensing information and meteorological data were combined to construct GSC, GPC, and GOC monitoring models for maize using the HLM method. Among all growth phases, the prediction effect improved obviously at maturity with *R* ^2^ increasing from 0.03 to 0.85 for GSC, from 0.14 to 0.87 for GPC, and from 0.21 to 0.50 for GOC. The best prediction accuracy during the whole growth period of each quality parameter, i.e., GSC, GPC, or GOC, also increased from 0.51, 0.49, and 0.36 to 0.90, 0.87, and 0.74, respectively (**Tables 4**, **7**). The main reason for the improvement was that remote sensing and meteorological data were used as double-nested structure data, the reciprocal influences between crop growth and environmental information on grain quality were considered, intra-annual and inter-annual errors could be corrected (**Figure 6**), and the scalability of the model was enhanced in this study. Although crop simulation models are key tools for assessing the effects of environmental factors on crop growth and development, most models still cannot simulate grain quality directly except for protein content (Nuttall et al., 2017). The HLM method used in this study provides an initial opportunity to extend the predictive power of maize grain quality characteristics.

### 4.2 Importance of meteorological data in predicting grain quality

Starch and protein accumulation in maize kernels have been reported to be regulated by water and temperature in previous studies (Singletary et al., 1994; Correndo et al., 2021; Guo et al., 2022). The sucrose required for starch accumulation was regulated by photosynthesis, which was affected by solar radiation, temperature, precipitation, and other meteorological factors. The status of nitrogen required for protein synthesis was thought to change dynamically in soil, affected by temperature and moisture (Archontoulis et al., 2014). Ali et al. (2010) found that grain oil content was also affected by water stress. In summary, the influence of weather data was considered when predicting and evaluating grain quality in many studies. Butts-Wilmsmeyer et al. (2019) evaluated the correlation between weather and grain quality at different stages using principal component analysis and found that temperature, precipitation, and maize grain quality were significantly correlated. Li et al. (2020) predicted GPC successfully using four weather parameters, i.e., average daily solar radiation, daily maximum and minimum temperature, and total precipitation 1 month prior to anthesis. Jahangirlou et al. (2023) also estimated grain starch, protein, and oil contents using crop models and logistic equations with detailed temperature and precipitation elements during the maize growth season.

In this study, three main meteorological data, i.e., *AT*, *Rad*, and *Pre*, were also used as the intra- and inter-annual variations to evaluate the grain quality under different growth stages. A strong relation was shown between meteorological factors and GSC, especially the deepest RD expressed between *Pre* and GSC at each growth stage, followed by *AT* (**Table 5**). Because of this, the prediction accuracy of GSC was improved greatly by HLM instead of MLR at various growth stages, especially at the maturation stage (**Table 7**). The relationship between weather data and GPC was weaker than the relationship between weather data and GSC. In addition, the good RD between weather data and GOC was only performed in the sowing–jointing stage. A similar performance also occurred in the accuracy change of the prediction model. In general, weather data, especially precipitation data, should be used as the main input parameters in the GSC prediction in the future, the comprehensive utilization of canopy spectral information and meteorological data can be used for the GSC and GPC prediction effectively. For the GOC prediction, more information should be considered to further improve the prediction accuracy.

### 4.3 Combination of prediction model under different growth stages

For the prediction of GSC, GPC, and GOC, the highest prediction accuracy showed in different growth stages. The best prediction result of GSC appeared at the grain-filling stage, followed by the tasseling and maturation stages. However, overfitting appeared in the prediction with the validation set at the tasseling and maturation stages (*R* ^2^ = 0.61 and 0.69) (**Table 7**). The best prediction result for GPC occurred at the grain-filling and maturation stages, while that for GOC occurred at the jointing stage. The rapid accumulation of starch and protein occurs from post-anthesis to grain-filling, while when starch deposition ceases, protein accumulation continues longer until near maturity (Vos, 1981), which may explain the difference in the optimal prediction period for GPC and GSC and the similar prediction accuracy for GPC at the grain-filling and maturation stages (*R* ^2^ = 0.84 and 0.87) (**Table 7**). However, the prediction results of GOC were completely different from those of GSC and GPC, which may be related to the high impact of meteorological data during an early stage. Unfortunately, most of the previous studies focused on the prediction under a single growth stage, and the combined prediction of multiple growth stages was scarce. We found that the accuracy of the prediction model under the combination of multiple growth stages, regardless of the modeling or validation set, was much higher than that under the single stage, indicating that the supplementary information of different growth stages was helpful to the monitoring of grain quality (**Tables 7**; **8**). In particular, the best prediction model of GSC in this study was the combination of predicted value under three phases, i.e., tasseling, grain-filling, and maturation (**Table 8**). However, predicted values at the tasseling and maturation periods account for a large proportion through the model coefficients, which is consistent with the result of Xie et al. (2020) on the monitoring of starch content in rice; that is, heading and maturity are the most suitable periods for predicting GSC.

Although we achieved good results in predicting grain quality in this study, the model was only established based on the phenological period of the single hybrid and the single experimental plot. When it was applied to other hybrids or sites in the future, hybrid and soil parameters that affect grain quality should be added to the model for prediction accuracy. However, too many parameters may limit the large-scale application of the model, and thus, the selection of parameters is extremely important.

## 5 Conclusions

The MLR model using only remote sensing data underestimated the interference of environmental factors when assessing maize grain quality. The problem of environmental deviation under different sowing dates and years was well-corrected based on HLM using hyperspectral and meteorological data. The accuracy of grain quality estimation was further improved, i.e., GSC (*R* ^2^ = 0.96, *RMSE* = 0.06, *MAE* = 0.05), GPC (*R* ^2^ = 0.90, *RMSE* = 0.09, *MAE* = 0.07), and GOC (*R* ^2^ = 0.85, *RMSE* = 0.10, *MAE* = 0.08), by combining the predicted values of HLM at different growth stages. These results showed a great potential to predict grain quality at both intra- and inter-annual scales in summer maize through the HLM method and the combination of multiple phases.

## Data availability statement

The original contributions presented in the study are included in the article/supplementary material. Further inquiries can be directed to the corresponding author.

## Author contributions

WS and QH conducted the investigation. WS, JL, XX, YW, SZ, SM, and RW implemented all the analyses. WS and QH conceptualized the study. WS prepared the original draft. JL, XX, YW, SZ, SM, and RW prepared sections of the manuscript. QH supervised the experiment. All authors contributed to the article and approved the submitted version.

## References

[B1] Ali Q. Ashraf M. Anwar F. (2010). Seed composition and seed oil antioxidant activity of maize under water stress. J. Am. Oil Chem. Soc 87, 1179–1187. doi: 10.1007/s11746-010-1599-5

[B2] Archontoulis S. V. Miguez F. E. Moore K. J. (2014). Evaluating APSIM maize, soil water, soil nitrogen, manure, and soil temperature modules in the midwestern united states. Agron. J. 106, 1025–1040. doi: 10.2134/agronj2013.0421

[B3] Asseng S. Milroy S. P. (2006). Simulation of environmental and genetic effects on grain protein concentration in wheat. Eur. J. Agron. 25, 119–128. doi: 10.1016/j.eja.2006.04.005

[B4] Butts-Wilmsmeyer C. J. Seebauer J. R. Singleton L. Below F. E. (2019). Weather during key growth stages explains grain quality and yield of maize. Agron. 9, 16. doi: 10.3390/agronomy9010016

[B5] Chen P. (2020). Estimation of winter wheat grain protein content based on multisource data assimilation. Remote Sens. 12, 3201. doi: 10.3390/agronomy9010016

[B6] Cho Y. Kang K. K. (2020). Functional analysis of starch metabolism in plants. Plants 9, 1152. doi: 10.3390/plants9091152 32899939 PMC7569781

[B7] Correndo A. A. Fernandez J. A. Vara Prasad P. V. Ciampitti I. A. (2021). Do water and nitrogen management practices impact grain quality in maize? Agron. 11, 1851. doi: 10.3390/agronomy11091851

[B8] Dash J. Curran P. J. (2007). Evaluation of the MERIS terrestrial chlorophyll index (MTCI). Adv. Space Res. 39, 100–104. doi: 10.1016/j.asr.2006.02.034

[B9] Dente L. Satalino G. Mattia F. Rinaldi M. (2008). Assimilation of leaf area index derived from ASAR and MERIS data into CERES-wheat model to map wheat yield. Remote Sens. Environ. 112, 1395–1407. doi: 10.1016/j.rse.2007.05.023

[B10] FAO (2020) (FAOSTAT). Available at: http://www.fao.org/faostat/en/#data/QC (Accessed July 2020).

[B11] Fitzgerald G. Rodriguez D. O’Leary G. (2010). Measuring and predicting canopy nitrogen nutrition in wheat using a spectral index–the canopy chlorophyll content index (CCCI). Field Crop Res. 116, 318–324. doi: 10.1016/j.fcr.2010.01.010

[B12] Gamon J. A. Penuelas J. Field C. B. (1992). A narrow-waveband spectral index that tracks diumal changes in photosynthetic efficiency. Remote Sens. Environ. 41, 35–44. doi: 10.1016/0034-4257(92)90059-S

[B13] Gao B. C. (1996). NDWI–a normalized difference water index for remote sensing of vegetation liquid water from space. Remote Sens. Environ. 58, 257–266. doi: 10.1016/S0034-4257(96)00067-3

[B14] Ghafoor A. Karim H. Asghar M. A. Raza A. Hussain M. I. Javed H. H.. (2021). Carbohydrates accumulation, oil quality and yield of rapeseed genotypes at different nitrogen rates. Plant Prod. Sci. 25 (1), 50–69. doi: 10.1080/1343943X.2021.1943464

[B15] Gooding M. (2017). The effects of growth environment and agronomy on grain quality (Cambridge: Woodhead Publishing).

[B16] Guo J. Qu L. Wei Q. Lu D. (2022). Effects of post-silking low temperature on the starch and protein metabolism, endogenous hormone contents, and quality of grains in waxy maize. Front. Plant Sci. 13. doi: 10.3389/fpls.2022.988172 PMC9673756 36407592

[B17] Huete A. Didan K. Miura T. Rodriguez E. P. Gao X. Ferreira L. G. (2002). Overview of the radiometric and biophysical performance of the MODIS vegetation indices. Remote Sens. Environ. 83, 195–213. doi: 10.1016/S0034-4257(02)00096-2

[B18] Jahangirlou M. R. Morel J. Akbari G. A. Alahdadi I. Soufizadeh S. Parsons D. (2023). Combined use of APSIM and logistic regression models to predict the quality characteristics of maize grain. Eur. J. Agron. 142, 126629. doi: 10.1016/j.eja.2022.126629

[B19] Jin X. Kumar L. Li Z. Feng H. Xu X. Yang G.. (2018). A review of data assimilation of remote sensing and crop models. Eur. J. Agron. 92, 141–152. doi: 10.1016/j.eja.2017.11.002

[B20] Khanal S. Fulton J. Shearer S. (2017). An overview of current and potential applications of thermal remote sensing in precision agriculture. Comput. Electron. Agric. 139, 22–32. doi: 10.1016/j.compag.2017.05.001

[B21] Kim H. Golub G. H. Park H. (2005). Missing value estimation for DNA microarray gene expression data: local least squares imputation. Bioinf. 21, 187–198. doi: 10.1093/bioinformatics/bth499 15333461

[B22] Koca Y. O. Erekul O. (2016). Changes of dry matter, biomass and relative growth rate with different phenological stages of corn. Agric. Agric. Sci. Proc. 10, 67–75. doi: 10.1016/j.aaspro.2016.09.015

[B23] Kuri F. Murwira A. Murwira K. S. Masocha M. (2014). “Predicting maize yield in Zimbabwe using dry dekads derived from remotely sensed vegetation condition index. Int. J. Appl. Earth Obs. Geoinf. 33, 39–46. doi: 10.1016/j.jag.2014.04.021

[B24] Li H. Fernie A. R. Yang X. (2023). Using systems metabolic engineering strategies for high-oil maize breeding. Curr. Opin. Biotechnol. 79, 102847. doi: 10.1016/j.copbio.2022.102847 36446144

[B25] Li Z. Taylor J. Yang H. Casa R. Jin X. Li Z.. (2020). A hierarchical interannual wheat yield and grain protein prediction model using spectral vegetative indices and meteorological data. Field Crop Res. 248, 107711. doi: 10.1016/j.fcr.2019.107711

[B26] Li Y. Wang P. Liu J. Zhang S. Li L. (2014). Evaluation of drought monitoring effects in the main growth and development stages of winter wheat using vegetation temperature condition index III-impact evaluation of drought on wheat yield. Agr. Res. Arid Areas 32 (5), 218–222. doi: 10.3390/su12072801

[B27] Li Z. Wang J. Xu X. Zhao C. Jin X. Yang G.. (2015). Assimilation of two variables derived from hyperspectral data into the DSSAT-CERES model for grain yield and quality estimation. Remote Sens. 7, 12400–12418. doi: 10.3390/rs70912400

[B28] Ma J. Zheng B. He Y. (2022). Applications of a hyperspectral imaging system used to estimate wheat grain protein: a review. Front. Plant Sci. 13. doi: 10.3389/fpls.2022.837200 PMC9024351 35463397

[B29] Maccioni A. Agati G. Mazzinghi P. (2001). New vegetation indices for remote measurement of chlorophylls based on leaf directional reflectance spectra. J. Photochem. Photobiol. B 61 (1), 52–61. doi: 10.1016/s1011-1344(01)00145-2 11485848

[B30] Marino S. Aria M. Basso B. Leone A. P. Alvino A. (2014). Use of soil and vegetation spectroradiometry to investigate crop water use efficiency of a drip irrigated tomato. Eur. J. Agron. 59, 67–77. doi: 10.1016/j.eja.2014.05.012

[B31] Ning T. Zheng Y. Han H. Jiang G. Li Z. (2012). Nitrogen uptake, biomass yield and quality of intercropped spring- and summer-sown maize at different nitrogen levels in the north China plain. Biomass Bioenergy 47, 91–98. doi: 10.1016/j.biombioe.2012.09.059

[B32] Nuttall J. G. O’Leary G. J. Panozzo J. F. Walker C. K. Barlow K. M. Fitzgerald G. J. (2017). Models of grain quality in wheat-a review. Field Crop Res. 202, 136–145. doi: 10.1016/j.fcr.2015.12.011

[B33] Onoyama H. Ryu C. Suguri M. Iida M. (2011). Estimation of rice protein content using ground-based hyperspectral remote sensing. Eng. Agric. Environ. Food 4 (3), 71–76. doi: 10.1016/S1881-8366(11)80015-7

[B34] Panigrahi N. Das B. S. (2018). Canopy spectral reflectance as a predictor of soil water potential in rice. Water Resour. Res. 54, 2544–2560. doi: 10.1002/2017WR021494

[B35] Paraginski R. T. Vanier N. L. Berrios J. D. J. de Oliveira M. Elias M. C. (2014). Physicochemical and pasting properties of maize as affected by storage temperature. J. Stored Prod. Res. 59, 209–214. doi: 10.1016/j.jspr.2014.02.010

[B36] Peñuelas J. Gamon J. A. Fredeen A. L. Merion J. Field C. B. (1994). Reflectance indices associated with physiological changes in nitrogen and water-limited sunflower leaves. Remote Sens. Environ. 48, 135–146. doi: 10.1016/0034-4257(94)90136-8

[B37] Pradawet C. Khongdee N. Pansak W. Spreer W. Hilger T. Cadisch G. (2022). Thermal imaging for assessment of maize water stress and yield prediction under drought conditions. J. Agron. Crop Sci. 00, 1–15. doi: 10.1111/jac.12582

[B38] Ranum P. Peña-Rosas J. P. Garcia-Casal M. N. (2014). Global maize production, utilization, and consumption. Ann. N.Y. Acad. Sci. 1312, 105–112. doi: 10.1111/nyas.12396 24650320

[B39] Rodrigues F. A. Blasch G. Defourny P. Ortiz-Monasterio J. I. Schulthess U. Zarco-Tejada P. J.. (2018). Multi-temporal and spectral analysis of high-resolution hyperspectral airborne imagery for precision agriculture: assessment of wheat grain yield and grain protein content. Remote Sens. 10, 930. doi: 10.3390/rs10060930 PMC7340494 32704487

[B40] Rondeaux G. Steven M. Baret F. (1996). Optimization of soil-adjusted vegetation indices. Remote Sens. Environ. 55, 95–107. doi: 10.1016/0034-4257(95)00186-7

[B41] Schlemmer M. Gitelson A. Schepers J. Ferguson R. Peng Y. Shanahan J.. (2013). Remote estimation of nitrogen and chlorophyll contents in maize at leaf and canopy levels. Int. J. Appl. Earth Obs. Geoinf. 25, 47–54. doi: 10.1016/j.jag.2013.04.003

[B42] Serrano L. Peñuelas J. Ustin S. L. (2002). Remote sensing of nitrogen and lignin in Mediterranean vegetation from AVIRIS data: decomposing biochemical from structural signals. Remote Sens. Environ. 81 (2), 355–364. doi: 10.1016/S0034-4257(02)00011-1

[B43] Shuai G. Basso B. (2022). Subfield maize yield prediction improves when in-season crop water deficit is included in remote sensing imagery-based models. Remote Sens. Environ. 272, 112938. doi: 10.1016/j.rse.2022.112938

[B44] Singletary G. W. Banisadr R. Keeling P. L. (1994). Heat-stress during grain filling in maize–effects on carbohydrate storage and metabolism. Aust. J. Plant Physiol. 21, 829–841. doi: 10.1071/PP9940829

[B45] Tan C. Wang J. Guo W. (2011). “Predicting grain starch content of winter wheat through remote sensing method based on HJ-1A/1B images,” in 2011 2nd International Conference on Artificial Intelligence, Management Science and Electronic Commerce (AIMSEC). 6303–6306. doi: 10.1109/AIMSEC.2011.6009646

[B46] Vaughan M. M. Block A. Christensen S. A. Allen L. H. Schmelz E. A. (2018). The effects of climate change associated abiotic stresses on maize phytochemical defenses. Phytochem. Rev. 17, 37–49. doi: 10.1007/s11101-017-9508-2

[B47] Vos J. (1981). Effects of temperature and nitrogen supply on post-floral growth of wheat: measurements and simulations (Wageningen: Pudoc).

[B48] Wang C. Feng M. Wang J. Xiao L. Yang D. (2013). Monitoring grain starch accumulation in winter wheat *via* spectral remote sensing. Chin. J. Eco-Agric. 21 (4), 440–447. doi: 10.3724/SP.J.1011.2013.00440

[B49] Wang L. Wang P. Li L. Xun L. Kong Q. Liang S. (2018). Developing an integrated indicator for monitoring maize growth condition using remotely sensed vegetation temperature condition index and leaf area index. Comput. Electron. Agric. 152, 340–349. doi: 10.1016/j.compag.2018.07.026

[B50] Wilson A. M. Silander J. A. Gelfand A. Glenn J. H. (2011). Scaling up: linking field data and remote sensing with a hierarchical model. Int. J. Geogr. Inf. Sci. 25, 509–521. doi: 10.1080/13658816.2010.522779

[B51] Wu C. Niu Z. Tang Q. Huang W. (2008). Estimating chlorophyll content from hyperspectral vegetation indices: modeling and validation. Agric. For. Meteorol. 148, 1230–1241. doi: 10.1016/j.agrformet.2008.03.005

[B52] Xie L. Wang F. Zhang Y. Huang J. Hu J. Wang F.. (2020). Monitoring of amylose content in rice based on spectral variables at the multiple growth stages. Trans. Chin. Soc Agric. Eng. 36 (8), 165–173. doi: 10.11975/j.issn.1002-6819.2020.08.020

[B53] Xu X. Teng C. Zhao Y. Du Y. Zhao C. Yang G.. (2020). Prediction of wheat grain protein by coupling multisource remote sensing imagery and ECMWF data. Remote Sens. 12, 1349. doi: 10.3390/rs12081349

[B54] Xue L. Cao W. Yang L. (2007). Predicting grain yield and protein content in winter wheat at different n supply levels using canopy reflectance spectra. Pedosphere 17 (5), 646–653. doi: 10.1016/S1002-0160(07)60077-0

[B55] Yue K. Li L. Xie J. Liu Y. Xie J. Anwar S.. (2022). Nitrogen supply affects yield and grain filling of maize by regulating starch metabolizing enzyme activities and endogenous hormone contents. Front. Plant Sci. 12. doi: 10.3389/fpls.2021.798119 PMC8847167 35185953

[B56] Zaman-Allah M. Vergara O. Araus J. L. Tarekegne A. Magorokosho C. Zarco-Tejada P. J.. (2015). Unmanned aerial platform-based multi-spectral imaging for field phenotyping of maize. Plant Methods 11, 35. doi: 10.1186/s13007-015-0078-2 26106438 PMC4477614

[B57] Zarco-Tejada P. J. Miller J. R. Mohammed G. H. Noland T. L. Sampson P. H. (2001). Scaling-up and model inversion methods with narrow-band optical indices for chlorophyll content estimation in closed forest canopies with hyperspectral data. IEEE Trans. Geosci. Remote Sens. 39 (7), 1491–1507. doi: 10.1109/36.934080

[B58] Zhang J. Song X. Jing X. Yang G. Yang C. Feng H.. (2022). Remote sensing monitoring of rice grain protein content based on a multidimensional euclidean distance method. Remote Sens. 14, 3989. doi: 10.3390/rs14163989

[B59] Zhao C. Liu L. Wang J. Huang W. Song X. Li C. (2005). Predicting grain protein content of winter wheat using remote sensing data based on nitrogen status and water stress. Int. J. Appl. Earth Obs. Geoinf. 7, 1–9. doi: 10.1016/j.jag.2004.10.002

[B60] Zhu Y. Yao X. Tian Y. Liu X. Cao W. (2008). Analysis of common canopy vegetation indices for indicating leaf nitrogen accumulations in wheat and rice. Int. J. Appl. Earth Obs. Geoinf. 10, 1–10. doi: 10.1016/j.jag.2007.02.006

